# Is ketamine and lamotrigine interactions responsible for the sub-therapeutic effect of ketamine?

**DOI:** 10.1192/j.eurpsy.2021.1060

**Published:** 2021-08-13

**Authors:** M. Adnan, F. Motiwala, Z. Mansuri, C. Trivedi, A. Reddy

**Affiliations:** 1 Psychiatry, Mercy Hospital and Medical Center, Lincolnwood, United States of America; 2 Psychiatry, Texas Tech University Health Sciences Center at the Permian Basin, Midland, United States of America; 3 Department Of Psychiatry, Boston Children’s Hospital/Harvard Medical School, Boston, United States of America; 4 Psychiatry, Psychiatry Austin, Austin, United States of America; 5 Psychiatry, Virginia Tech Carilion School of Medicine, Roanoke, United States of America

## Abstract

**Introduction:**

The immediate antidepressant effect of Ketamine has become a breakthrough in the treatment of depression. Cytochrome CYP3A4 and 2B6 primarily metabolize Ketamine.

**Objectives:**

The present study explores potential pharmacokinetic and pharmacodynamic interactions of Lamotrigine and Ketamine.

**Methods:**

A literature search was conducted using (“ketamine” OR “Lamotrigine” AND Interactions in PubMed, Embase, and PsycINFO. Our literature search resulted in 72 hits and result in qualified five studies.

**Results:**

We found five studies: one RCT study, a RCT, a crossover design, Two case reports, and one murine model study. In the first RCT conducted on 16 healthy normal volunteer subjects. lamotrigine significantly decreased ketamine-induced perceptual abnormalities (P < 0.001), positive (P < 0.001) and negative symptoms (P < 0.05), and learning and memory impairment (P < 0.05) which shows the counter effect of ketamine. Another study revealed Ketamine evoked increases in all the BPRS subscale scores, and all scores were lower after lamotrigine pretreatment. A case report from 2014 reports the failure of ketamine anesthesia in a patient with lamotrigine overdose. Another case report mentions that Lamotrigine reduced the craving in a patient with ketamine use disorder. A murine model study with lamotrigine showed improved PPI (Prepulse inhibition) ketamine-induced disruption. These results suggest that Lamotrigine may exert this effect via a glutamatergic system.

**Conclusions:**

The literature review suggests that Lamotrigine interferes with glutamatergic neurotransmission reducing the effect of Ketamine. It is not clear how this may impact Ketamine’s antidepressant action. Future large scale and well-designed RCTs are required to confirm these findings.

**Conflict of interest:**

No significant relationships.

